# Exploring New Frontiers: A Cross-Sectional Analysis of Explosive Phase and Muscle Activation During Maximal Biting in Women with Temporomandibular Disorder and Orofacial Pain

**DOI:** 10.3390/medsci13040306

**Published:** 2025-12-06

**Authors:** Bianca Rossi Botim, Mayra Evelise Cunha dos Santos, Arthur Ferreira Esquírio, Kariny Realino do Rosário Ferreira, Ana Clara Leal, Gabriel Alves Godinho, Maria de Cássia Souza Macedo, Thaís Carvalho Oliveira, Gabriela Lopes Gama, Michelle Cristina Sales Almeida Barbosa, Alexandre Wesley Carvalho Barbosa

**Affiliations:** Musculoskeletal Research Group—NIME, Department of Physical Therapy, Federal University of Juiz de Fora, Av. Moacir Paleta 1167, Governador Valadares 36036-900, MG, Brazil; bianca.botim@estudante.ufjf.br (B.R.B.); mayra.evelise@estudante.ufjf.br (M.E.C.d.S.); arthurferreira.esquirio@estudante.ufjf.br (A.F.E.); kariny.realino@estudante.ufjf.br (K.R.d.R.F.); anaclara.leal@estudante.ufjf.br (A.C.L.); gabriel.godinho@estudante.ufjf.br (G.A.G.); mariacassia.macedo@estudante.ufjf.br (M.d.C.S.M.); thais.carvalho@estudante.ufjf.br (T.C.O.); gabriela.gama@ufjf.br (G.L.G.); michellecsalmeida@ufjf.br (M.C.S.A.B.)

**Keywords:** temporomandibular disorders, electromyography, pre-activation, rate of force development

## Abstract

**Background:** Temporomandibular disorders (TMDs) are associated with altered masticatory muscle function and pain. Although electromyographic parameters have been extensively studied, the rate of force development (RFD) remains an underexplored biomarker in this context. **Objective:** Analyze the RFD differences in women diagnosed with and without TMD. As a secondary outcome, the masseter and temporalis muscle pre-activation values were compared between groups based on the biting force onset. Additionally, neuromuscular efficiency analysis was also performed. **Methods:** A retrospective analysis of 62 medical records (41 with TMD, 21 controls) was conducted. Electromyographic activity and bite force were measured during three 5-s maximal biting tasks using synchronized surface electromyography (sEMG) and a laboratory-grade load cell. RFD was computed from force–time curves. Muscle pre-activation was assessed based on sEMG activity immediately preceding contraction onset. **Results:** The TMD group showed a significantly smaller RFD (mean = 85.5 N/s) compared to controls (mean = 109.0 N/s; *p* = 0.03; Cohen’s d = 0.5). No significant differences were found in neuromuscular efficiency and pre-activation or post-activation levels of the masseter and temporalis muscles between groups. **Conclusions:** RFD distinguishes women with TMD from healthy controls and may represent a sensitive biomechanical marker of neuromuscular adaptation in TMD, although confirmatory studies are needed. The absence of neuromuscular efficiency and pre-activation differences suggests compensatory neuromuscular mechanisms. Further prospective studies are needed to validate these findings and explore clinical applications.

## 1. Introduction

The temporomandibular joint (TMJ) is one of the most frequently moved joints in the human body [1,2]. The TMJ is very active during functional tasks, such as chewing and speech [1,2], comprising a complex rotational-translational mandibular movement [1,3]. Movement at one side necessarily affects the opposite [3]. As the right and left TMJs move simultaneously, biomechanical issues may occur in both TMJs and their structures (cartilage, synovial membrane), resulting in musculoskeletal and neuromuscular disorders (temporomandibular disorders or TMDs) [4,5].

Similarly to other chronic pain conditions, TMD is more prevalent in female working-aged people [5,6,7]. The prevalence ranges from 5 to 12% globally, with a multifactorial etiology [1,7]. Some of the major factors contributing to TMD incidence are the bruxism occurrence and emotional factors, which may also contribute to orofacial and cervical muscle hyperactivity [7,8,9]. Individuals with TMD often experience reduced quality of life [3,7] and limitations in chewing and communication [3,6,7].

TMD is the leading cause of orofacial pain [4,6,10], with two subgroups according to their characteristics: those with joint-related symptoms and the muscle-related subgroup, the most frequent subtype, with symptoms on the masticatory muscles [10]. The muscular TMD has a prevalence of up to 13% in adult women [11] and is related to variations in the capacity to produce force during the processes of electrical excitation [12,13,14]. Furthermore, elevated and sustained muscle activity in the masseter may be related to tissue hypoxia, pain, and fatigue [15].

Fatigue is a frequently present symptom in the TMD. Masticatory muscles affected by TMD and fatigue typically exhibited increased activation amplitude, reflecting the recruitment of extra motor units, along with a shift in the mean power frequency, which is consistent with a decline in muscle fiber conduction velocity [16,17,18], a parameter measured through surface electromyography (sEMG) [16]. The sEMG is a non-invasive method based on recording the myoelectric signals to estimate the motor units’ temporospatial summation [19,20]. In this sense, sEMG is capable of measuring the electrical potential generated by muscle fibers and is often used to assess the masticatory muscle electrophysiological behavior and function in patients with TMD [19,21,22].

Furthermore, sEMG is frequently used as a measure of response to therapeutic approaches, comparison between TMD subgroups, and a diagnostic aid [20,23,24]. In this sense, sEMG can be used as a measure of the masticatory motor adaptation [22,25]. These adaptations to a behavior or muscle state may reflect the potential for neuroplasticity of areas of the sensorimotor cortex [8,25,26]. These adaptations can be functional but also dysfunctional, which may induce or intensify clinical manifestations such as pain [8,25,26]. In this sense, the formation of action potentials in painful contractions can be altered, as well as the electromyographic record [12,19,27].

TMD has a complex diagnostic process [28] based on biopsychosocial, functional and structural characteristics [29]. However, marked biomechanical and neuromuscular changes are expected in a chronic myogenic disorder such as TMD [30]. Some variability may occur among individuals experiencing the disorder [19,30,31], although similarities are greatly expected as the physiological response is essentially the same in specific groups [19,32]. In this sense, to objectively set the diagnostic outcome, biomarkers are often the major choice. Recent studies highlighted some biochemical targets as potential biomarkers correlated to TMD symptoms [1,29,33,34,35]. Evidence also reports some masticatory muscle functional indexes using the sEMG as a diagnostic approach [23,36]. In this sense, Nickel et al. (2024) also reports the need to develop new biomarkers to assess TMD [37]. These same authors suggested a new biomarker based on mechanobehavioral scoring, assessed through sEMG [37].

Furthermore, the sEMG has also been used to estimate the muscle excitation onset [38]. To assist motor performance, some studies reported that anticipatory mechanisms coordinate changes in basal muscle excitation previously to any voluntary movement [39,40]. In the orofacial musculature, the effector muscles are also responsible for stabilizing and controlling movements [41,42]. Several studies have investigated the sEMG amplitude and signal variations in different tasks [19,28,43], but no studies have analyzed the muscle excitation timing in individuals with TMD compared to healthy individuals.

Furthermore, in patients with TMD, changes in the masticatory motor unit’s recruitment can compromise muscle functionality, although these changes are being investigated due to the lack of consensus [12,13]. Neuromuscular efficiency (NME) is an index that represents the ability to generate force relative to the level of muscular excitation [44,45]. The NME is calculated through the ratio between the amount of neural stimulus and the force generated by a muscle [46,47]. Based on the reported neuromuscular and functional changes, it would be reasonable to assume that the NME would be altered in the TMD population compared to healthy cohorts.

In this sense, it has been proposed that functional and coordinating alterations of the masticatory muscles would underlie the symptoms of TMD [25]. Recent reviews have found several TMD masticatory muscles’ functional changes compared to healthy controls [13,19,43]. Those changes appear to vary according to the task performed (rest, clenching, chewing) as well as the muscles analyzed [43]. However, the direct association between TMD and masticatory disorders is not yet a general consensus [13].

Neuromuscular excitation and the motor unit recruitment pattern are also determinants of another parameter not yet fully explored considering the masticatory muscles: the rate of force development (RFD) [48,49,50]. The RFD is calculated as the ratio between the variation in force and the time during the muscle contraction [51]. RFD is derived from force–time or torque-time curves recorded during explosive voluntary contractions [48,51,52]. Explosive strength is defined by Maffiuletti et al. (2016) as the ability to increase force or torque as quickly as possible during a rapid voluntary contraction, starting from a low contraction or resting level [51]. The RFD is used to characterize explosive strength [51,53]. Performance in sports and functional tasks is highly correlated to RFD [53], as it exhibits high sensitivity in detecting acute and chronic changes in neuromuscular function, eliciting distinct physiological mechanisms compared to the maximum voluntary contraction [31,49,51,54].

However, to the best of our knowledge, no studies were found verifying the masseter and temporalis muscles’ RFD in TMD patients. Therefore, the present study aimed to analyze the RFD differences in women diagnosed with and without TMD. As a secondary outcome, the masseter and temporalis muscle pre-activation values were compared between groups based on the biting force onset. Additionally, neuromuscular efficiency analysis was also performed and reported. The primary hypothesis is that women diagnosed with TMD would exhibit a significantly lower RFD of the masticatory muscles during maximal biting compared with healthy controls. This reduction is expected to reflect impairments in neuromuscular performance and altered motor unit recruitment related to chronic orofacial pain. The secondary hypothesis is that the NME would be significantly lower in the TMD group, indicating decreased ability to translate neural excitation into mechanical output. Finally, the tertiary hypothesis is that the pre-activation timing of the masseter and temporalis muscles would differ between women with and without TMD. Specifically, the TMD group was expected to show delayed or less synchronized pre-activation, reflecting altered anticipatory control and motor coordination.

## 2. Materials and Methods

### 2.1. Participants

This study was carried out based on a retrospective data analysis of the medical records database from the Musculoskeletal Research Group. These data came from a previous study at the Physiotherapy School Clinic at Governador Valadares (Minas Gerais, Brazil), conducted between January 2018 and December 2018 and approved by the Federal University of Juiz de Fora (UFJF) Ethics Committee Board under the protocol number CAAE: 68457617.6.0000.5147 (Date of approval: 26 July 2024). The present retrospective study was also approved by the same Ethics Committee under the protocol number CAAE: 81392424.3.0000.5147. The total sample consisted of 102 medical records. Those with missing or inconsistent data were excluded, resulting in a total of 62 actual medical records to be analyzed.

In the 2018 study, the participants were recruited by public invitation through flyers and personal contacts. The Research Diagnostic Criteria for Temporomandibular Disorders (RDC/TMD-Axis I) was used for TMD diagnoses. The RDC/TMD is the internationally accepted gold standard, and its most recent version is the DC/TMD [55]. However, the lack of a validated Portuguese version of the DC/TMD during data collection led the previous authors to use the validated Portuguese version of the RDC/TMD. Only those with chronic TMD (more than 6 months of complaints) were included. All participants had myalgia according to the RDC/TMD. Inclusion criteria for both groups were having at least 28 permanent teeth and an age range of 18 to 45 years [55]. All participants reported no periodontal problems. Exclusion criteria for both groups were as follows: history of facial and/or TMJ trauma; osteoarthritis; confirmed pain attributable to migraine, headache or neck pain; recent or chronic use (more than 6 months) of any analgesic, anti-inflammatory or psychiatric medication; acute infection or other significant disease of the teeth, ears, eyes, nose or throat; and presenting any neurological or cognitive deficit [55]. Data on age and body mass index (BMI) were available for all included participants. The groups did not differ significantly in age (*p* = 0.64) or BMI (*p* = 0.57). Written informed consent has been obtained from all participants to publish this paper.

### 2.2. Equipment

The excitation of temporal and masseter muscles during maximal isometric biting was assessed using an acquisition module with eight analog channels (Miotec™, Biomedical Devices, Porto Alegre, RS, Brazil). An A/D board performed the conversion from analog to digital signals with a 16-bit resolution input range, a sampling frequency of 2 kHz, a common rejection module greater than 100 dB, a signal–noise ratio of less than 3 μV Root Mean Square, and an impedance of 109 Ω. The collected data was windowed at 125 ms using the Miotec™ Suite Software Version 1.0. The sEMG signals were recorded in root mean square in μV with surface Meditrace™ (Ludlow Technical Products, Gananoque, ON, Canada) Ag/AgCl electrodes with a diameter of 1 cm and center-to-center distance of 1 cm, applied in a transverse orientation parallel to the underlying fibers on a muscle site. A reference electrode was placed on the left lateral humeral epicondyle. The sEMG signals were amplified and filtered (Butterworth fourth-order, 20–450 Hz bandpass filter, 60 Hz notch filter). All pieces of information were recorded and processed using the software Miotec Suite™ (Miotec Biomedical Devices, Porto Alegre, RS, Brazil). Before sEMG electrode placement, the skin was cleaned with 70% alcohol, followed by an exfoliation using a specific sandpaper for the skin and a second cleaning with alcohol. The electrodes were positioned on the anterior temporal muscles and the superficial masseter on both the left and right sides parallel to the muscle fibers according to a previous study [55].

### 2.3. Experimental Protocol

**Figure 1:**
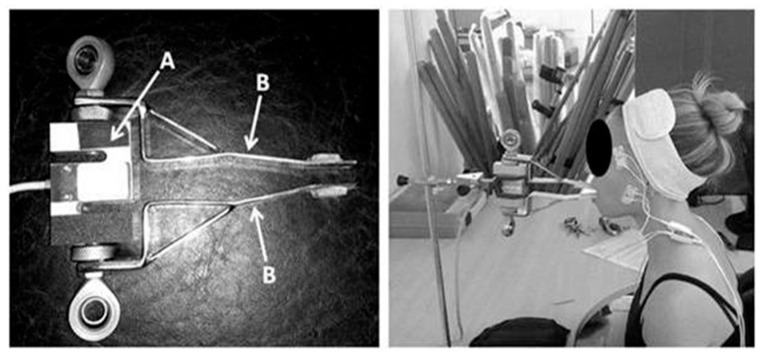
Adapted load cell. (**A**) laboratory-grade load cell; (**B**) adapted arms [56].

Three 5-s maximum isometric biting tasks (MIBTs) were performed by each participant while biting on an adapted load cell (Miotec™, Biomedical Devices, Porto Alegre, RS, Brazil; maximum tension-compression = 200 Kgf, precision of 0.1 Kgf, maximum error of measurement = 0.33%). Five seconds were previously collected during rest to establish task onset. Each MBIT was followed by 5 min of rest. The participants were asked to sit comfortably (the volunteer remained seated with the trunk erect, feet on the floor, and hands resting on the thighs) while the load cell’s arms were positioned on the incisors (Figure 1). A disposable material was used to cover the load cell’s arms for each participant. The forward head posture was controlled during all procedures by positioning the load cell closer to the participants so they could bite in their natural head posture. Standardized verbal commands (“start,” “keep biting,” “stop”) were used by the same rater for all tests’ recordings. A 5-s familiarization was followed by 3 min of rest before the MBIT. The load cell was coupled and synchronized with the acquisition module [55].

### 2.4. Data Extraction

**Figure 2:**
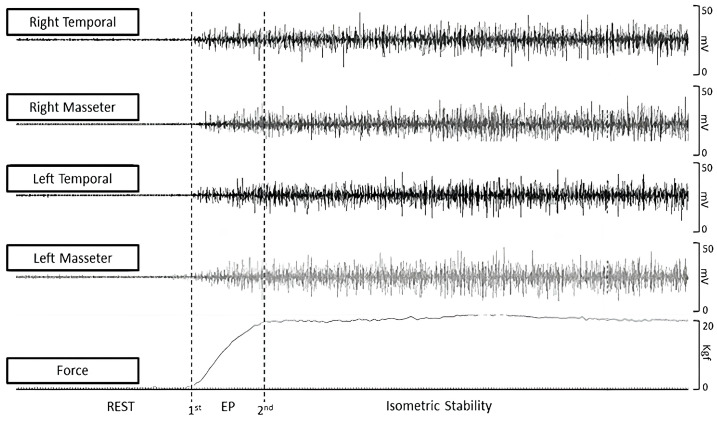
Force and sEMG data extraction. EP = explosive force.

All data were extracted offline using Miotec Suite™ software (Miotec™, Biomedical Devices, Porto Alegre, RS, Brazil). Two markers were used to differentiate the explosive and isometric phase windows (Figure 2). As the load cell was synchronized with the electromyography channels, the first marker was at the onset of the force. Three 1-s windows of the rest period with 1 s of separation between them were collected, and the onset of the force was defined by three times the standard deviation of the average resting intervals plus the mean itself. Three 1-s isometric stability windows with 1 s of separation between them were collected, and the second marker was defined by three times the standard deviation of the average isometric stability interval minus the mean itself (Figure 2). The force onset was detected when the signal exceeded three standard deviations above the mean resting baseline. RFD was then computed as the first derivative of the force–time curve (ΔForce/ΔTime) over the 0–1 s explosive window following onset. This 1-s interval corresponded to the “explosive phase” used consistently across all trials, while the subsequent 1-s window represented the “isometric phase.” Each participant performed three trials, and the average RFD across the three trials was used for analysis. The raw force signal was low-pass filtered (Butterworth, 10 Hz, 4th order) before differentiation to minimize noise.

Muscle activation onset was determined from the EMG signal exceeding three standard deviations above the mean baseline within a 200 ms window preceding or following the force onset. Pre-activation: EMG onset < 0 ms relative to force onset. Normal activation: EMG onset within 0–50 ms after force onset. Post-activation: EMG onset > 50 ms after force onset.

The dependent between-group variables were as follows: (1) Peak load (the maximum force output achieved during the MIBT); (2) Mean explosive load (the mean force output during the explosive phase); (3) Mean isometric load (the mean force output during the isometric phase); (4) The sEMG (right and left masseter and temporalis muscles); (5) Right and left neuromuscular efficiency (neuromuscular efficiency was defined as the ratio of the sEMG to the mean explosive force output).

### 2.5. Raters

An independent rater (rater 1) conducted the inclusion and exclusion process for the participants as previously described. Two additional raters (2 and 3) received extensive training before the study to perform data extraction using the Miotec software, following the windowing method previously detailed above. The prior training was essential to ensure the procedure’s reliability and minimize human error during data extraction. Another independent rater (rater 4) conducted the statistical analysis.

### 2.6. Statistical Analysis

Descriptive data were presented as mean and standard deviation. Normality and homogeneity were assessed separately for each group using the Shapiro–Wilk and Levene tests, respectively. Based on the results of the normality of the RFD data, between-group comparisons (with and without TMD) were performed using the Student’s *t*-test for independent samples for bite force and the Mann–Whitney test for independent samples for muscle excitation. When Levene’s test indicated unequal variances, Welch’s *t*-test was applied. Neuromuscular efficiency (NME) was defined as the ratio between the magnitude of muscle activation and the corresponding mechanical output, expressed as normalized EMG amplitude divided by force (EMG_norm/Force). The EMG amplitude was normalized to the maximal compound activity obtained during the calibration contraction and expressed as a percentage of the compound maximal voluntary contraction (% CIVM). The resulting NME values therefore represent the relative neural drive required to produce a given level of bite force, with higher values indicating lower mechanical efficiency (i.e., greater excitation per unit of force). This formulation was intentionally adopted to emphasize the neural recruitment effort associated with force generation, consistent with our study’s objective to characterize neuromuscular activation strategies rather than mechanical efficiency per se. Units are expressed as % of the CIVM times the force in Newtons (%·N^−1^). The responsiveness of the predictive measures was assessed by the chi-square test (X^2^) to establish the association between the pre- and post-activation assessments in individuals with and without TMD. Additionally, Cramér’s V was used to measure the strength of association between categorical variables. Given the exploratory nature and limited number of comparisons, we did not apply formal multiplicity corrections. For the analysis of neuromuscular efficiency, considering the results of data normality, comparisons between the groups were conducted using the Mann–Whitney test. Statistical significance was defined as *p* < 0.05. Cohen’s d was used to verify the effect size of the interventions between the groups, qualitatively classified as <0.2: trivial effect; 0.2–0.5: small effect; 0.5–0.8: medium effect; ≥0.8: large effect. The analysis was performed using JAMOVI software (The JAMOVI project, version 2.3.21, retrieved from: http://www.jamovi.org).

## 3. Results

**Figure 3:**
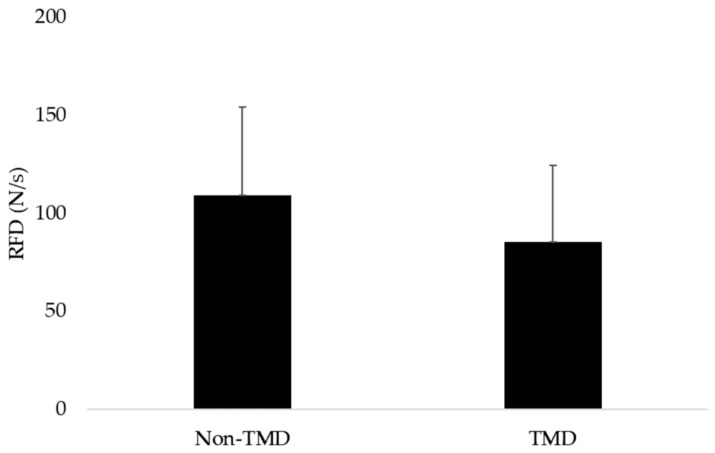
Mean and standard deviation values of groups with and without TMD.

The RFD descriptive data are summarized as follows: The group with TMD (n = 41) showed a mean RFD of 85.5 N/s, with a standard deviation of 38.8 N/s. In contrast, the group without TMD (n = 21) showed a mean of 109 N/s, with a standard deviation of 45.2 N/s (Figure 3).

Each participant performed three maximal biting trials separated by 5 min rest. For each dependent variable (RFD, EMG, NME), the mean of the three trials was used for analysis. To assess reliability, we calculated intra-class correlation coefficients (ICC [3,1]) and coefficient of variation (CV%) for RFD across the three trials: ICC = 0.91 (95% CI = 0.86–0.94), CV = 7.8%, indicating excellent within-session reliability. The *t*-test for independent samples revealed a significant difference in the RFD between the groups with and without TMD (*p* = 0.03), with a Cohen’s d of 0.5, indicating a moderate effect size, with the confidence interval excluding zero (95% CI: 0.0067 to 1.12).

In the pre-activation and NME analyses, two missing data were found in RT and one missing data in RM. In the descriptive analysis of neuromuscular efficiency, data are summarized as follows: The group without TMD showed a median of 0.04%.N^−1^, (range: 0.02–0.26%.N^−1^) for LM, 0.05%.N^−1^, (range: 0.01–0.10%.N^−1^) for RM, 0.05%.N^−1^, (range: 0.01–0.38%.N^−1^) for LT, and 0.08, (range: 0.02–0.32%.N^−1^) for RT. In contrast, the group with TMD showed a median 0.04%.N^−1^, (range: 0.01–0.60%.N^−1^) for LM, 0.04%.N^−1^, (range: 0.01–0.33%.N^−1^) for RM, 0.07%.N^−1^, with a range from 0.01 to 0.77%.N^−1^ for LT, and 0.08%.N^−1^, with a range from 0.01 to 0.61%.N^−1^ for RT. The Mann–Whitney test found no between-group NME differences for LM (*p* = 0.50), RM (*p* = 0.59), LT (*p* = 0.53), or RT (*p* = 0.73) during the explosive force period.

Finally, no between-group differences were found for electromyographic pre- and post-activation and regular activation, as shown in Table 1.

## 4. Discussion

The results suggest that the RFD analysis showed a significant between-group difference, with the TMD group showing lower values, with a moderate effect size. No between-group differences were observed for pre- and post-activation for any analyzed muscle (LM, RM, LT, RT). Furthermore, contingency table analyses and chi-square tests did not reveal any significant association between the presence of TMD and muscle activation patterns. No between-group differences were observed for NME for any muscle (LM, RM, LT, RT).

Several methods have been described to diagnose TMD, but accuracy has always been an issue [19,23,57]. In this context, functional masticatory muscle indexes emerge as promising markers to support the accurate TMD characterization, although confirmatory studies are still needed [23,36]. RFD has been suggested as a marker to characterize some neuromuscular conditions, such as neuromuscular fatigue [49]. RFD is modulated by neural mechanisms, depending on the reaction of motor units at the beginning of contraction [49,50]. In this sense, the increase in muscle activation in the initial phase of contraction is notably important to provoke a rapid force production [48,50,51]. Furthermore, reviews report that RFD may represent a more efficient concept in identifying dysfunctions or functional adaptations in the neuromuscular system [31,49,51], similar to those produced by muscle damage or pain [58,59], when compared to maximum voluntary strength [49,51], supporting the current analysis in the context of TMD.

Additionally, compared to the maximum voluntary contraction force parameter, RFD was more strongly correlated to the functional tasks [51,60]. It is known that individuals with TMD may exhibit impaired masticatory neuromuscular function [13,61,62], which, based on neurophysiology, could be reflected in RFD. Therefore, assuming this parameter may also be altered in individuals with TMD would be reasonable. However, to our knowledge, this is the first study to investigate RFD in a muscular TMD sample. In this sense, reviewing the literature, Rodríguez-Rosell et al. (2018) discussed the physiological factors correlated to RFD and reported that even in homogeneous groups, rapid force production differs between individuals, suggesting that the mechanisms involved in explosive contractions may vary significantly [31]. This variability appears to occur due to variations in the speed at which the nervous system recruits motor units, a parameter known as the motor unit recruitment rate [48,50,63]. Furthermore, the firing rate of motor neurons also appears as one of the mechanisms responsible for modulating RFD [48]. However, several methods for obtaining and analyzing RFD are also related to a notable variability in its values [31,51].

However, it is often expected that in the presence of pain, RFD will be reduced [58]. Despite the absence of studies that investigated this parameter in individuals with TMD, it is possible to infer its decrease from other contexts in which there is also the presence of pain and RFD reduction. In this sense, a study investigated the effects of acute induced pain on maximal force production and RFD of the quadriceps during isometric muscle activation [58]. The authors determined the RFD as the average slope of the curve, and its analysis was carried out in segmented time intervals. [58]. In contrast, the current study differed methodologically by analyzing RFD over only one total time interval. The authors observed both a reduction in RFD and a reduction in the rate of increase in EMG. Furthermore, the impact of pain on RFD differed between time periods, which may be attributed to the close relationship between initial force development (<75 ms) and the maximum motor unit discharge rate [48,50], which may be reduced during tasks involving acute nociceptive activation [58]. In the current study, RFD was also shown to be reduced in the symptomatic group; however, given the methodological and anatomical differences, comparisons are limited.

Another cross-sectional study compared RFD in women with patellofemoral pain and physically active healthy women. RFD was calculated as the slope of the torque-time curve, measuring the muscle mass-normalized torque variation. RFD and maximal torque were reduced in the pain group, with RFD showing greater deficits compared to strength. However, when analyzing muscle thickness of hip abductors and extensors, no significant differences were observed between the groups. The authors concluded that changes in RFD should not be explained by muscle thickness or the relative amount of non-contractile tissue, but rather by mechanisms involving neuromuscular changes [64].

In situations where time to develop strength is limited, explosive strength is essential [65]. The precise movements during chewing are related to the masseter’s ability to develop force, even quickly [66,67]. Explosive force is characterized by RFD [51]. However, several factors within the neuromuscular system influence RFD [51]. In the context of TMD, pain [18,19,68] and muscle imbalance [13,62,69] are prominent characteristics, which may be associated with the RFD reduction observed in the symptomatic group. In this sense, the influence of pain on masticatory muscle activity was previously described according to different theories and models of pain explanation [70,71]. According to Peck et al. (2008), the Vicious Cycle Theory suggests that pain leads to muscle hyperactivity, which in turn causes more pain. However, this theory presents little solid evidence, giving rise to the Pain Adaptation Model, which predicts inhibition of agonist muscles and increased activity of antagonists. However, this model was also considered insufficient to elucidate the complexity of the relationship between pain and motor activity [71].

Subsequently, with the aim of proposing an alternative to theories that proposed only excitation or inhibition of muscular activity, Minami et al. (2013) investigated masseter muscle activity under induced pain conditions and observed a complex reorganization of the electromyographic activity [27], including both inhibition and recruitment of new motor units, as well as varied changes in the firing rate of active units [27]. These changes may reflect a motor control strategy so that the force produced is not significantly impacted during pain [27]. These findings are consistent with previous descriptions, which also report changes in the angle of force during pain [72]. Furthermore, Sohn et al. (2000) previously demonstrated a decrease in the firing rate of masseter motor units at low and moderate force levels, although isometric force and recruitment threshold remained constant during induced pain in the masseter [73].

However, these studies use experimental models of acute pain induction [27,74], which involve mechanisms distinct from those of chronic pain and do not take into account psychosocial factors such as stress and anxiety [74], changes in behavior or damage to musculoskeletal integrity [58], frequently present in chronic TMD [75]. Furthermore, chronic pain mechanisms can modulate pain sensitivity, contributing to bruxism and greater masticatory tension [76,77]. In this sense, the psychosocial state can affect neuromuscular function, and in the long term, induce changes in muscle morphology [76]. In this sense, using an animal model of chronic stress, Pereira et al. (2019) found histopathological changes in the masseter, including irregular changes in the diameters of muscle fibers, as well as irregularity in their contours [78]. Ispir et al. (2022) used ultrasound to verify the variation in masseter thickness and its relationship with parafunctional habits. [79]. The authors did not observe significant differences attributed to these habits, although they found great variability between individuals according to vertical facial morphology [79]. Furthermore, a recent study observed greater median stiffness in the masseter during contraction performed by individuals with myofascial pain and TMD compared to healthy controls [80]. However, the effects of muscle architecture and muscle stiffness on RFD are still unclear, although increases in area and volume may be related to a greater absolute rate of strength increase [51]. 

In addition to rapid muscle activation mediated by neural factors, RFD also has muscular determinants, such as muscle fiber type and composition [31,51,81]. The characteristics of motor units are manifested through a heterogeneous composition of muscle fibers and myosin proteins. These characteristics are remarkably important for the chewing process [28,66,67]. Fast fatigable fibers are activated for tasks that require high strength, accompanied by speed [66]. However, the simultaneous presence of different myosin isoforms is associated with greater variability in both contractile strength and speed [66,67]. This unique composition allows the muscle to modulate its contraction force to optimize total energy expenditure, distinguishing it from other skeletal muscles [66,67].

Furthermore, several studies demonstrate that the masticatory muscles of individuals with TMD present reduced oxygen extraction [15,82,83,84]. This change may be due to parafunctions such as sustained clenching or overloading during chewing, which is responsible for interrupting blood flow, contributing to a more anaerobic environment. [15,82,83]. This hypoxic condition can favor the accumulation of metabolites such as lactate and H^+^ [85], impacting energy production [82,83]. This situation can impair masticatory muscle control, in addition to requiring greater metabolic and energy demand for functional activities, which may be accompanied by fatigue, pain [82,83] and hyperalgesia. [85]. Furthermore, the nervous system opts for muscle recruitment strategies that provide the best mechanical advantage with the least energy expenditure [72]. However, when contractions are painful, this recruitment may become less efficient in preventing further harmful stimuli [72]. Although the present study did not measure masticatory muscle oxygenation, based on this pathophysiology, it would be rational to propose that the NME would be altered in participants with TMD. Despite this, the present study did not find significant differences in the NME of participants with chronic TMD.

Often, the NME index is obtained through the relationship between the quantity of neural stimuli and the muscular force generated [45,46], through sEMG and dynamometry [45]. In this sense, the index can assume motor functioning, from neural command to its relative force production [45,86]. In the literature related to TMD, the terminology “neuromuscular efficiency index” is not easily found, although similar variations such as “muscular efficiency” [13,55] and “functional efficiency” [12,13] are extensively described. Furthermore, several studies that investigated neuromuscular changes in TMD analyzed concepts similar to those present in the index [12,19,87]. In this sense, a study found a positive correlation between maximum bite force and EMG activity of the masseter and temporalis in people with TMD; however, they found no significant differences in sEMG activity for any of the muscles analyzed between the TMD and control groups [12]. Furthermore, findings such as reduction in electromyographic signal during sustained maximum squeezing [19] or reduction in maximal force [86] could lead to a reduction in neuromuscular efficiency. However, based on the EMG/force relationship, it would also be reasonable to propose that an increase in electrical activity, possibly related to motor compensation strategies [12], accompanied or not by an increase in the force produced, could reflect an apparently normal index.

Furthermore, methodological variations regarding data collection and analysis are also observed in the index [44,45,46,47]. Likewise, procedures for measuring masticatory efficiency are also diverse [13], although most studies agree that individuals with TMD have impaired orofacial motor functions [13,25]. In this sense, it is also possible that the time period for extracting sEMG and strength data may have influenced the capture of metabolic and neuromuscular changes reflected in the NME, which demonstrates the need for new studies that assess the NME index in individuals with TMD compared to healthy cohorts.

The effective production of mandibular movement occurs through the coordinated activation of the masticatory muscles [41,88]. Furthermore, together with the other structures of the stomatognathic system, these muscles act on the dynamic stability of the TMJ [41,42]. In this sense, sensorimotor integration is responsible for the refined control of masticatory muscle activation [87]. However, nociceptive stimuli can modulate this function [86,88]. The literature reports that, between the moment of onset of muscle excitation and the beginning of force production, there is a physiological electromechanical delay [89]. However, in painful conditions, muscle excitation may develop late [89]. Although studies assessing muscle timing in individuals with TMD are scarce, several studies demonstrate changes in parameters such as force production and control [87], magnitude of muscle activation [19,28], and symmetry and muscle coordination indices [25,28]. Based on this, it would be reasonable that the timing of muscle activation would be altered in the presence of TMD. Although the current study did not find significant differences in this variable, other studies reinforce the hypothesis of changes in muscle timing in the presence of pain associated or not with the absence of muscle integrity [90,91,92].

Furthermore, despite the absence of significant differences in muscle timing between groups, RFD showed reduced values in the TMD group. Dieterich et al. (2017) analyzed the temporal relationship between estimates of the onset of muscle activation and physical movement, recorded by sEMG and ultrasound, respectively [89]. One of the findings was that a higher rate of torque development correlates with a significant reduction in regional variation in EMG onset, as well as a reduction in the time intervals between EMG onset and movement onset [89].

Some limitations must be addressed. First, as a retrospective design, it relies on pre-existing data, which precludes direct control over variables that may not have been initially recorded. Additionally, the Portuguese version of the RDC/TMD, rather than the more recent DC/TMD version, represents another limitation. While the RDC/TMD is widely recognized, the current diagnostic standard is the DC/TMD, and its use would have been preferable for consistency with contemporary diagnostic criteria. The sample consisted solely of individuals aged 18 to 45 with at least 28 permanent teeth. Unmeasured factors such as occlusion and facial morphology may impair generalization. This limitation restricts the generalization of the findings to other age groups or individuals with varying dental characteristics. As a result, it may introduce selection bias and reduce the study’s external validity. Furthermore, the absence of studies that analyzed the same variables was a factor that limited the comparisons made.

The present findings provide initial evidence that the RFD may serve as a clinically relevant biomarker of masticatory neuromuscular performance in women with temporomandibular disorders. Beyond distinguishing symptomatic individuals from healthy controls, RFD can offer a dynamic measure of functional recovery throughout rehabilitation. Because RFD reflects the efficiency and speed of motor unit recruitment, it is particularly sensitive to neuromuscular adaptations that occur following therapeutic interventions such as masticatory muscle endurance training, occlusal splint therapy, or non-invasive neuromodulation approaches. In this sense, monitoring RFD before and after treatment may complement traditional clinical outcomes—such as pain intensity and EMG amplitude—by providing an objective, quantitative indicator of improvement in motor control and muscle coordination.

## 5. Conclusions

This study sought to identify RFD as a significant differentiator between women with and without TMD and to provide a novel biomechanical insight into the neuromuscular alterations associated with the disorder. Although significant differences were found, future research should validate these findings in diverse populations and explore their diagnostic applications. Furthermore, new studies conducted with different collection and analysis methodologies must be carried out in order to confirm or refute the other findings.

## Figures and Tables

**Table 1:** Pre- and post-activation and normal activation results.

Muscle	Group	Pre-Activation n (%)	Post-Activation n (%)	Normal Activation n (%)	*p*	Cramér’s V
LM	With TMD	7 (58.3%)	16 (59.3%)	18 (78.3%)	0.30	0.19
	Without TMD	5 (41.7%)	11 (40.7%)	5 (21.7%)		
RM	With TMD	8 (61.5%)	17 (65.4%)	15 (68.2%)	0.92	0.05
	Without TMD	5 (38.5%)	9 (34.6%)	7 (31.8%)		
LT	With TMD	10 (76.9%)	15 (65.2%)	16 (61.5%)	0.62	0.12
	Without TMD	3 (23.1%)	8 (34.8%)	10 (38.5%)		
RT	With TMD	8 (80.0%)	14 (58.3%)	17 (65.4%)	0.48	0.15
	Without TMD	2 (20.0%)	10 (41.7%)	9 (34.6%)		

Legend: LM = Left masseter, RM = Right masseter, LT = Left temporal, RT = Right temporal. All chi-square tests met assumptions; all expected cell frequencies were ≥ 5, and no standardized residuals were > 2.0.

## Data Availability

The data presented in this study are openly available in Mendeley Data at DOI: 10.17632/65cmtpd77y.1.
